# The Promoter of the Cereal *VERNALIZATION1* Gene Is Sufficient for Transcriptional Induction by Prolonged Cold

**DOI:** 10.1371/journal.pone.0029456

**Published:** 2011-12-29

**Authors:** Maria M. Alonso-Peral, Sandra N. Oliver, M. Cristina Casao, Aaron A. Greenup, Ben Trevaskis

**Affiliations:** 1 Division of Plant Industry, The Commonwealth Scientific and Industrial Research Organisation, Canberra, Australian Capital Territory, Australia; 2 Department of Genetics and Plant Production, Aula Dei Experimental Station, Consejo Superior de Investigaciones Científicas, Zaragoza, Aragón, Spain; University of Melbourne, Australia

## Abstract

The *VERNALIZATION1* (*VRN1*) gene of temperate cereals is transcriptionally activated by prolonged cold during winter (vernalization) to promote flowering. To investigate the mechanisms controlling induction of *VRN1* by prolonged cold, different regions of the *VRN1* gene were fused to the *GREEN FLUORESCENT PROTEIN* (*GFP*) reporter and expression of the resulting gene constructs was assayed in transgenic barley (*Hordeum vulgare*). A 2 kb segment of the promoter of *VRN1* was sufficient for *GFP* expression in the leaves and shoot apex of transgenic barley plants. Fluorescence increased at the shoot apex prior to inflorescence initiation and was subsequently maintained in the developing inflorescence. The promoter was also sufficient for low-temperature induction of *GFP* expression. A naturally occurring insertion in the proximal promoter, which is associated with elevated *VRN1* expression and early flowering in some spring wheats, did not abolish induction of *VRN1* transcription by prolonged cold, however. A translational fusion of the promoter and transcribed regions of *VRN1* to *GFP*, VRN1::GFP, was localised to nuclei of cells at the shoot apex of transgenic barley plants. The distribution of VRN1::GFP at the shoot apex was similar to the expression pattern of the *VRN1* promoter-*GFP* reporter gene. Fluorescence from the VRN1::GFP fusion protein increased in the developing leaves after prolonged cold treatment. These observations suggest that the promoter of *VRN1* is targeted by mechanisms that trigger vernalization-induced flowering in economically important temperate cereal crops.

## Introduction

Plants growing in temperate regions time flowering to coincide with favourable seasonal conditions. Winter frost can damage cold sensitive reproductive organs, while heat and water stress during summer can reduce fertility, so in temperate regions many plants flower in spring when conditions are optimal. One cue that promotes spring flowering is prolonged exposure to winter cold - vernalization [1]. Vernalization-induced flowering is a feature of many plants, including Arabidopsis and economically important temperate cereal crops, such as wheat (*Triticum spp.*) and barley (*Hordeum vulgare*), although different genes mediate this seasonal flowering response in these distantly related angiosperms (see [2]).

The *VERNALIZATION1* gene (*VRN1*) is a central regulator of vernalization-induced flowering in temperate cereals (see [2]–[5]). *VRN1* encodes a MADS box transcription factor that promotes flowering [6]–[9]. *VRN1* is transcribed at low basal levels but transcript abundance increases with prolonged cold treatment [6]–[9]. The response of *VRN1* expression to cold is quantitative, with longer cold treatments inducing higher transcript levels [6], [7], [10], [11]. This parallels the degree to which flowering is accelerated [11]. Mutants that lack *VRN1*, and flanking genes, are unable to flower [12], [13]. *VRN1*-like genes are likely to play similar roles in other temperate grasses, including economically important species such as *Lolium perenne* and *Phleum pratense* [14], [15].

Vernalization activates expression of *VRN1* in the leaf and shoot apex. At the shoot apex expression of *VRN1* promotes the transition to reproductive development [11], [16], [17]. Expression of *VRN1* in leaves unlocks the long-day flowering response, allowing long daylengths to further accelerate reproductive development post-vernalization [3], [16]. Molecular analyses have identified potential regulatory targets of *VRN1*. These include the grass/cereal specific transcription factors *VRN2* and *ODDSOC2* [18]–[21].

Alleles of *VRN1* that are expressed without cold treatment allow flowering without vernalization [6]–[8]. These “active alleles” have been used to breed wheats and barleys that flower without vernalization (spring types), which are grown where vernalization does not occur. Some active alleles of the wheat *VRN1* gene have mutations near the transcriptional start site, which might disrupt promoter sequences required to repress transcription prior to winter [8], [22], [23]. Additionally, the first intron of *VRN1* contains a broad region that is required to maintain repression of *VRN1* prior to winter [10], [24]–[27]. Alleles lacking large sections of the first intron are actively expressed and are associated with early flowering without vernalization, whereas some alleles lack smaller segments of the first intron and are associated with moderate increases in *VRN1* activity and weaker promotion of flowering [26], [27]. An insertion of a mobile genetic element at the 5′ end of the first intron is also associated with active expression of *VRN1* without vernalization [28], so the repressive action of the first intron is not related simply to size.

The state of chromatin at the *VRN1* locus appears to be an important determinant of activity [29]. Without vernalization, the chromatin at *VRN1* has high levels of the repressive histone modification histone 3 lysine 27 tri-methylation (H3K27Me3), which is typically associated with an inactive chromatin state [29]. This modification is found within the first intron of the *VRN1* gene and at the start point of transcription, sites that are critical for repression [29]. The presence of H3K27Me3 at these sites might contribute to repression of *VRN1* prior to winter [29].

The mechanisms that activate expression of *VRN1* in response to prolonged cold are unclear. Although the floral repressor *VRN2* is required to delay flowering prior to vernalization [18], cold induction of *VRN1* takes place in conditions where *VRN2* is not actively expressed and can occur in the absence of the *VRN2* gene [11], [16], [19]. So *VRN2* seems unlikely to mediate cold-induction of *VRN1*. The hypothesis that the *VEGETATIVE TO REPRODUCTIVE TRANSITION 2* gene is repressed by cold to allow increased expression of *VRN1* [30] also seems unlikely, since *VRT2* expression increases at low temperatures [31]. Furthermore, although *VRT2* binds to a sequence motif at the promoter of *VRN1 in vitro,* this motif is not critical for low-temperature induction [23]. Finally, alleles of *VRN1* that lack most of the first intron, which are expressed at high basal levels, are induced by cold [27]. This suggests that cold activation might occur independently of the repressive mechanism that acts at the first intron [27].

Studies in Arabidopsis, and other plants, have demonstrated that activation of transcription by low temperatures can be mediated by *cis*-acting elements located in the promoters of low-temperature responsive genes [32]. In this study, we examine whether the promoter of the *VRN1* gene can mediate a transcriptional response to prolonged cold, using transgenic barley as a model system.

## Results

### A *VRN1* promoter reporter gene fusion is expressed in the leaves and shoot apex of transgenic barley plants

**Figure 1:**
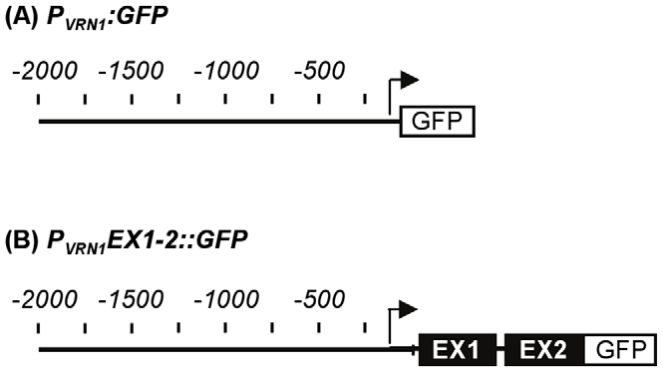
Schematic representation of *VRN1*reporter gene constructs. (A) A 2.0 kb fragment (SpeI-NcoI) from the *VRN1* promoter was fused to the GFP reporter gene with the *NOPALINE SYNTHASE (NOS)* terminator sequence to generate the *P_VRN1_:GFP* construct. (B) The *P_VRN1_EX1-2:GFP* construct was generated by fusing the same promoter region to exon1 and exon 2 of *VRN1* (EX1 and EX2), separated by a 0.35 kb segment of intron 1, followed by the *GFP-NOS* cassette. Scale indicates base pairs from the translational start site of MADS box open reading frame, indicated as negative relative to the coding regions of the *VRN1* locus. Arrow indicates transcriptional start site, which did not vary between control or prolonged cold treatments.

**Figure 2:**
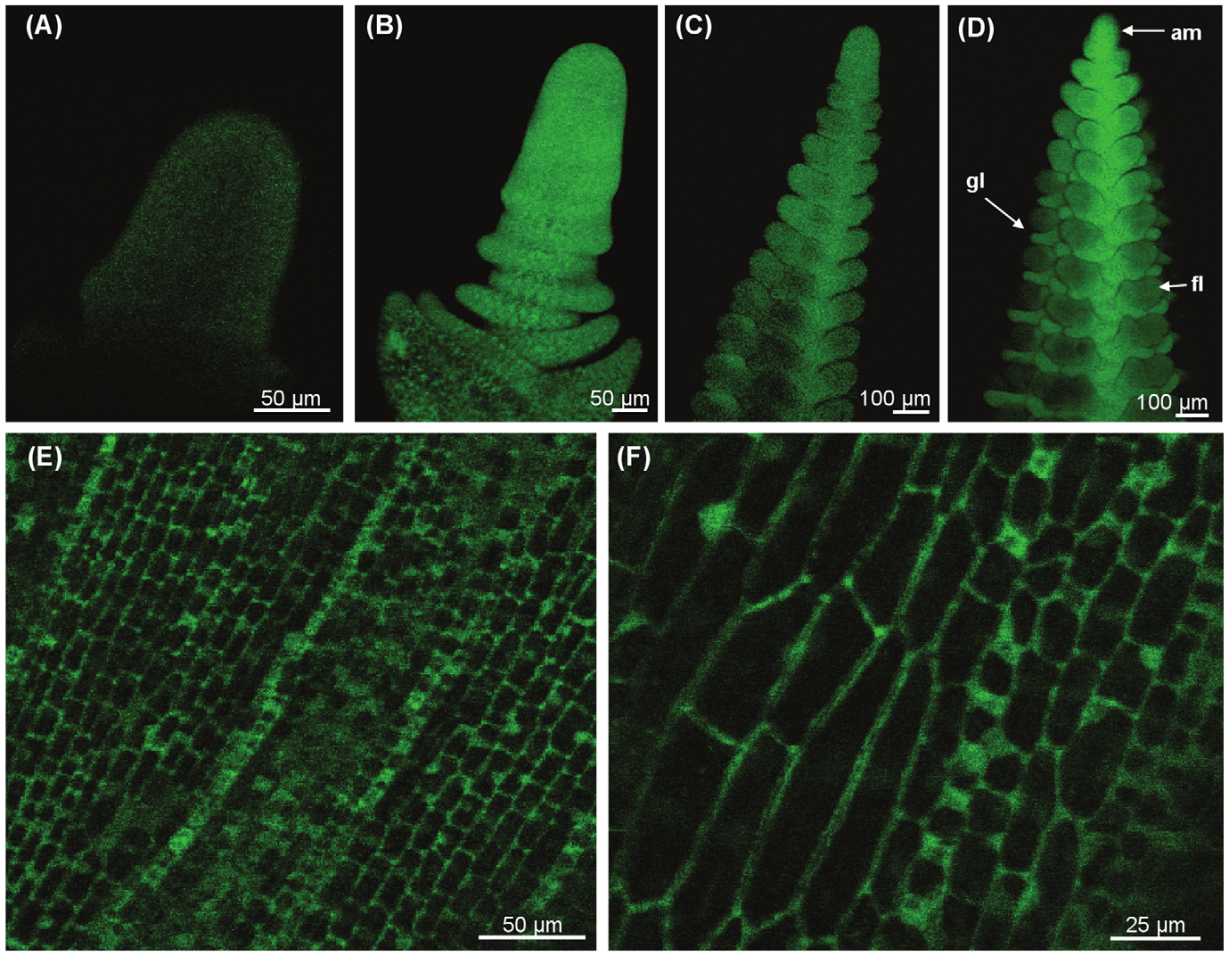
GFP fluorescence in the leaves and shoot apices of transgenic plants with the *P_VRN1_:GFP* construct. (A) GFP fluorescence in the vegetative shoot apex of 12 day old transgenic plants carrying the *P_VRN1_:GFP* construct. (B) The shoot apex at the late vegetative stage of 18 day old plants. (C,D) Shoot apices at different stages of reproductive development. (E,F) Fluorescence in the cytoplasm of cells in the developing leaves of 18 day old plants. am indicates the apical meristem, fl indicates the developing florets, gl indicates glume primordia.

A 2 kb fragment from the promoter of the barley *(Hordeum vulgare) VERNALIZATION1* gene (referred to hereafter as *VRN1*) was fused to the *GFP* reporter gene (Figure 1A) and the resulting construct *(P_VRN1_:GFP)* was transformed into barley (cv. Golden Promise). GFP activity was detected in the shoot apex and developing leaves of transgenic barley plants that carry the *P_VRN1_:GFP* construct (Figure 2). GFP fluorescence increased at the shoot apex at the late vegetative stage (Figure 2B) and remained active throughout the shoot apex during reproductive development, although expression was lower in the developing florets than in other parts of the inflorescence (Figure 2D). GFP fluorescence was also observed in leaves (Figure 2E and 2F).

### The promoter of *VRN1* is sufficient to mediate transcriptional induction of the *GFP* reporter gene by prolonged cold

**Figure 3:**
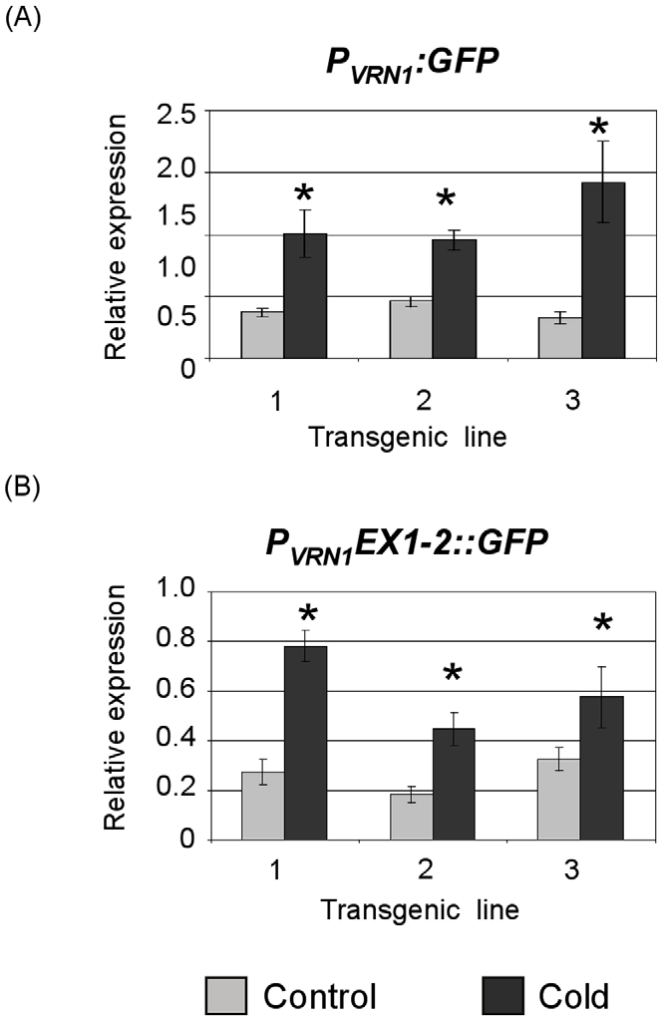
Expression of the *P_VRN1_:GFP* and *P_VRN1_EX1-2::GFP* constructs in control versus cold treated seedlings. (A) GFP transcript levels in transgenic seedlings carrying the *P_VRN1_:GFP* construct. Expression was assayed by quantitative reverse transcriptase PCR in control seedlings, germinated in darkness at normal glasshouse temperatures (20 degrees for 5 days), compared to seedlings germinated and grown in darkness to an identical stage of development at low temperatures (4 degrees for 28 days). Expression levels were assayed in three independent transgenic lines. (B) Expression of the *P_VRN1_EX1-2::GFP*, assayed as outlined above, in three independent transgenic lines. Expression is shown relative to *ACTIN*. Error bars show standard error for a minimum of 3 biological replicates. * indicates P<0.05.

The levels of GFP transcript generated from the *P_VRN1_:GFP* construct were assayed by quantitative reverse transcriptase PCR (qRT-PCR) in transgenic seedlings exposed to prolonged cold treatment (28 days at 4°). Expression was assayed in three independent transgenic lines. Transcript levels were higher in seedlings at the end of prolonged cold treatment than in control seedlings grown to an identical stage of development at normal temperatures (4 days at 20°) (Figure 3A). The degree of induction by cold varied between 1.5 to 4 fold, depending on the transgenic line assayed.

A second reporter gene fusion was constructed by fusing the same 2 kb region of the *VRN1* promoter to the *GFP* reporter gene, with the addition of the first and second exons from the *VRN1* gene, separated by a small segment (0.35 kb) of the first intron *(P_VRN1_EX1-2::GFP,* Figure 1B *)*. The spliced version of this translational fusion construct was expressed in transgenic barley plants, although no GFP fluorescence was detected, possibly due to poor translation or instability of the fusion protein produced. Levels of the spliced *P_VRN1_EX1-2::GFP* transcript increased in response to prolonged cold treatment (Figure 3B), similar to the *P_VRN1_:GFP* construct.

### Cellular and tissue localisation of a *VRN1::GFP* translational fusion in transgenic barley

**Figure 4:**
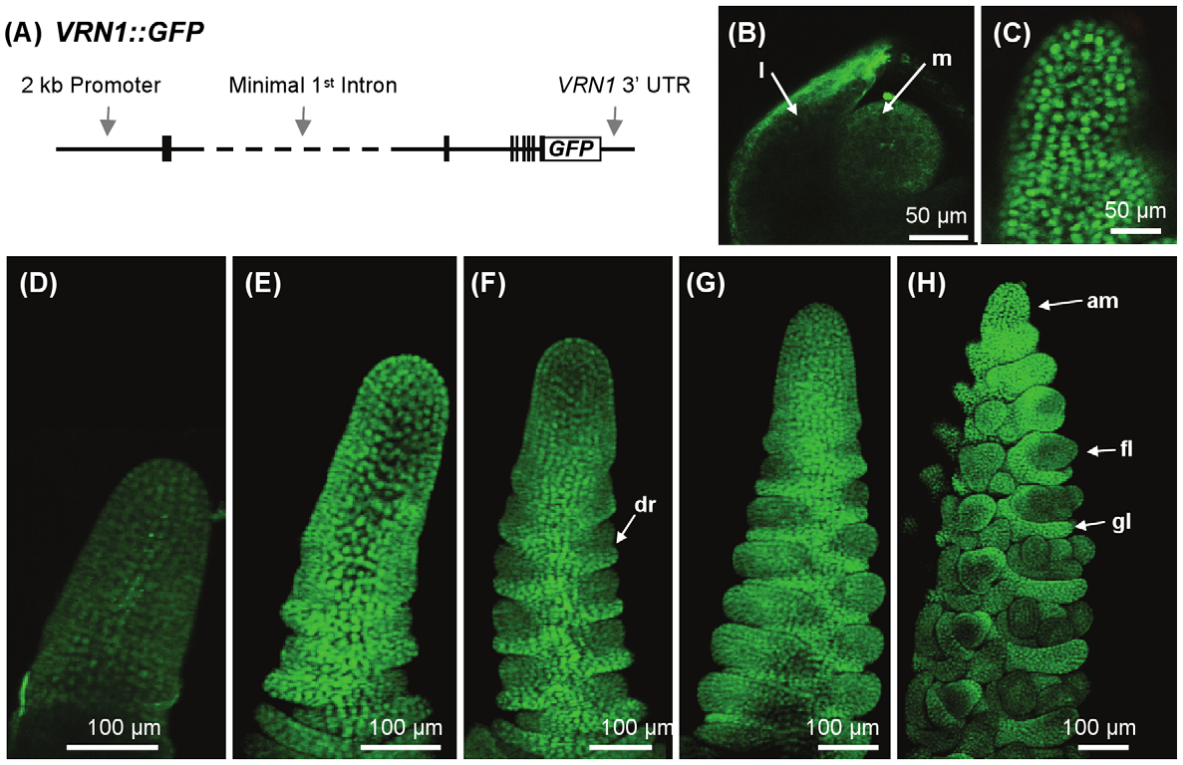
Activity of a VRN1::GFP fusion protein in transgenic barley plants. (A) Schematic representation of a *VRN1::GFP* fusion construct that fuses the entire *VRN1* gene, minus most of the first intron, to the GFP reporter gene followed by the 3′ UTR and terminator sequence of the *VRN1* gene. Vertical bars represent exons, dotted line represents the missing segment from intron 1, relative to the wildtype version of *VRN1* from vernalization responsive barleys (B) Low signal from the VRN1::GFP fusion in the vegetative shoot apical meristem (m) and developing leaf (l) of 7 day old seedlings. (C) Nuclear localisation of the VRN1::GFP fusion protein in the meristem of a reproductive shoot apex. (D–H) Time course of shoot apex development showing vegetative shoot apices (D,E), a shoot apex at the transition to reproductive development (F), indicated by double ridges (dr), and reproductive shoot apices (G,H). am indicates the apical meristem, fl indicates the developing florets, gl indicates glume primordia. Images B–H were taken on identical settings to allow direct comparison of fluorescence levels. (note: image C is a section from image H, 2 fold expanded).

**Figure 5:**
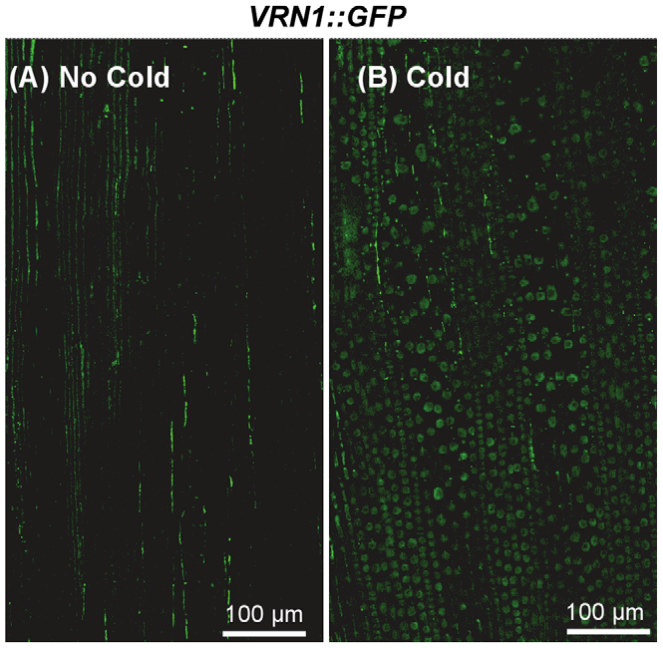
Activity of a VRN1::GFP fusion protein in the developing leaves of transgenic barley plants. (A) Low signal from the VRN1::GFP fusion in the developing leaves of plants grown at normal glasshouse temperatures. (B) Nuclear localisation of the VRN1::GFP fusion protein in cells within the developing leaves of seedlings germinated and grown at low temperatures to an identical stage of development. Images were taken with identical settings to allow comparison of fluorescence levels. Similar results were seen in two independent transgenic lines. Some background signals, “green lines” caused by reflection, are present in both images.

The *VRN1* gene, the promoter and transcribed regions minus the majority of the large first intron (1.1 kb of the 10.7 kb intron was present in the construct), was translationally fused to the *GFP* reporter gene followed by the 3′ UTR of the *VRN1* gene. The resulting *VRN1::GFP* construct (Figure 4A) was transformed into barley. GFP fluorescence signal was observed in the nuclei of cells (Figure 4). The nuclear VRN1::GFP fluorescence increased during vegetative shoot apex development and was higher in the pre-double ridge and double ridge apices than in the early vegetative shoot apex (Figure 4). At later stages of development, the VRN1::GFP signal was detected throughout the shoot apex, although expression was lower in the developing florets than in other parts of the inflorescence, similar to the *P_VRN1_:GFP* construct (Figure 4H). *VRN1::GFP* transcript levels increased in seedlings during prolonged cold treatment, although induction was weaker than that observed in the *P_VRN1_:GFP* lines (Figure S1). Whereas GFP signal was weak in the developing leaves of seedlings grown without cold treatment, strong fluorescence was observed in the nuclei in cells from the developing leaves of seedlings that had experienced prolonged cold (Figure 5).

### Cold response motifs and a small upstream open reading frame are found in the promoter and 5′ untranslated region of the *VERNALIZATION1* gene

**Figure 6:**
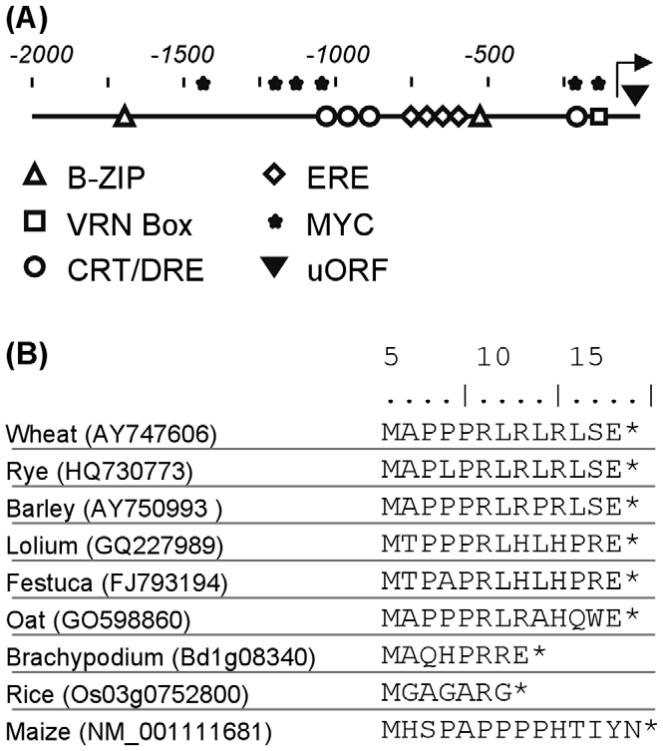
Potential regulatory motifs at the promoter of the *VRN1* locus. (A) Schematic representation of potential transcription factor binding sites at the promoter of *VRN1*. ERE indicates potential ethylene response element (GCCGCC), CRT/DRE: C-repeat transcription factor core binding site (CCGAC), MYC: MYC transcription factor binding site (CANNTG), B-ZIP: B-ZIP transcription factor binding site (ACGT). VRN Box: indicates the position of the putative vernalization regulatory motif suggested by Pidal *et al.* [23]. uORF denotes the position of the small upstream open reading frame. Scale indicates bp from transcriptional start site, which is indicated by an arrow. (B) Alignment of the predicted amino acid sequences of the upstream open reading frame from the 5′ untranslated regions of *VRN1* genes from temperate cereals (wheat, rye, barley, oats), temperate grasses (*Lolium, Festuca, Brachypodium*) and *VRN1* orthologues from warm climate cereals (rice and maize). Scale indicates amino acid residues from initiation codon.

The promoter of *VRN1* was scanned for potential *cis*-acting elements, by searching for sequence motifs similar to known transcription factor binding sites. This identified putative binding sites for *C-REPEAT BINDING FACTOR (CBF)* and *INDUCER OF CBF1 (ICE1)* transcription factors within the 2 kb of sequence upstream of the predicted translational start site at the *VRN1* gene (Figure 6A, Figure S2). These transcription factors have been shown to activate the expression of cold-responsive genes in Arabidopsis, and other plants [32]. Binding sites for B-ZIP and ethylene responsive transcription factors were also identified.

Another potential regulatory element identified at the *VRN1* locus is a small upstream open reading frame (uORF) encoding 13 amino acids (MAPPPRLRPRLSE) in the predicted 5′ leader sequence of the *VRN1* mRNA (Figure 6B). As predicted, the uORF is found in the 5′ end of *VRN1* cDNA sequences in GENBANK, and this was further confirmed by 5′ RACE (Rapid Amplification of cDNA ends). The transcriptional start site, which did not vary between vernalized versus non-vernalized plants is annotated in Figure S2. The presence of a proline rich small open reading frame is a conserved feature of the *VRN1* genes of barley and wheat. uORFs are also a feature of *VRN1*-like genes from other cereals and grasses, upstream of the main open reading frame that encodes the *AP1*/*FRUITFULL*-like MADS box transcription factor (Figure 6B). Small uORFs can influence translation from downstream ORFs by decreasing translational initiation rates or by stalling ribosomes and causing non-sense mediated message decay (see [33]).

### An insertion in the VRN box does not abolish induction of *VRN1* by prolonged cold

**Figure 7:**
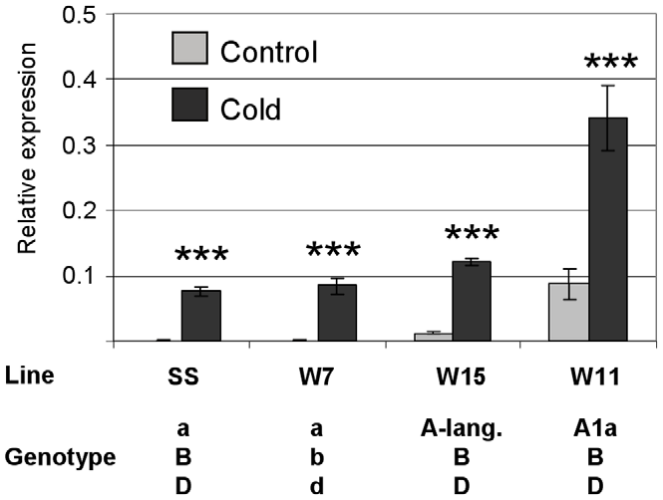
Transcript levels for the *VRN1* gene (A genome) of wheat near-isogenic lines with different *VRN1* genotypes. Transcript levels of the A genome copy of *VRN1* were assayed in hexaploid bread wheat (*Triticum aestivum*) near-isogenic lines that vary for *VRN1* genotype. Expression was assayed by quantitative reverse transcriptase PCR, using A genome specific primers, in seedlings germinated in darkness at 20° for 5 days (control) or 4° for 6 weeks (cold). The wheat lines used carry different combinations of wildtype *VRN1* alleles on each of the three genomes (a, b or d) or alleles with high basal activity (*A1a*, *A-lang.*, B or D). The *A1a* allele (line W11) has a promoter insertion in the VRN box of the *VRN1* gene on the A genome, but a full-length first intron [22]. The *A-lang.* allele (line W15), first identified in the Langdon cultivar of the tetraploid wheat *Triticum durum* [24], has a 7.2 kb deletion within the first intron, but has the wildtype promoter sequence. Similarly, the B and D alleles have deletions within the first intron of the *VRN1* genes on the B and D genomes respectively [24]. Expression is shown relative to *ACTIN*, error bars show standard error for four biological replicates, *** indicates P<0.001.

Mutations in a proximal promoter region defined as the VRN box are associated with elevated *VRN1* expression and early flowering in wheat (Pidal *et al.* 2009). To examine whether mutations in the VRN box influence the capacity for low-temperature induction of *VRN1*, transcriptional induction of *VRN1* by prolonged low-temperature treatment was examined in near-isogenic wheat lines with different alleles of the *VRN1* gene: a wildtype allele on the A genome (normal VRN box, full length first intron, parental line) versus alleles with either a 7,222 bp deletion within the first intron (line W15) or a 231 bp insertion in the VRN box on the A genome (line W11). Basal expression from the *VRN1* gene on the A genome (*VRN-A1*) was highest in the W11 line carrying the promoter insertion allele (Figure 7), and was also elevated in the W15 line carrying the intron deletion allele (Students T-tests: P<0.05, P<0.01 versus the parental line with the wildtype allele, for the promoter insertion or intron deletion alleles respectively). Low-temperature induction of the A genome copy of *VRN1* occurred irrespective of *VRN1* genotype (Figure 7). Thus, mutations that are associated with increased basal expression of the *VRN1* gene copy on the A genome (homeoallele) do not prevent low-temperature induction.

## Discussion

The 2 kb of DNA sequence upstream of the primary translational start site (MADS box open reading frame) of the *VRN1* gene is sufficient to drive expression of the *GFP* reporter gene in the shoot apex and leaves; organs where *VRN1* expression has been detected using quantitative RT-PCR [8], [11], [17]. The distribution of *P_VRN1_:GFP* expression observed in shoot apices and leaves of barley (Figure 2) is similar to the expression patterns of the *VRN1* genes of wheat (*T. aestivum*, *T. monococcum*) and oats (*Avena sativa*) detected by *in situ* hybridisation [17], [30]. Similarly, at later stages of barley inflorescence development (Figure 2D), the expression pattern of the *P_VRN1_:GFP* construct is consistent with the results of *in situ* hybridisation analysis of *VRN1* expression, which show expression of *VRN1* in meristems and organ primordia of the developing barley spike [34].

Expression of the *P_VRN1_:GFP* and *VRN1::GFP* reporter genes increased at the shoot apex shortly before the transition to reproductive development (inflorescence initiation). This is consistent with the observation that *VRN1* transcript levels increase at the shoot apex of this barley cultivar prior to inflorescence initiation, when assayed by quantitative RT-PCR [31]. Similarly, when the promoter of the maize (*Zea mays*) orthologue of *VRN1 (ZmMADS4)* was fused to the *β-GLUCURONIDASE* reporter gene an increase in reporter activity occurs at the equivalent stage of development in transgenic maize plants [35]. This expression pattern was confirmed by *in situ* hybridisation [35]. Thus, the expression patterns of the *VRN1* promoter reporter genes used in this study are similar to those of the endogenous *VRN1* genes from a range of cereals.

The increase in *VRN1* expression prior to inflorescence initiation and the distribution of *VRN1* expression in the developing inflorescence are consistent with a role for *VRN1* in promoting inflorescence meristem or organ identity. The lower *VRN1* activity seen in the developing florets (Figure 2D) suggests that less *VRN1* activity is required for the differentiation of floral organs. Other *VRN1-like* genes, *Barley MADS3* (*BM3)* and *BM8*, might contribute to the development of these organs, compensating for lower *VRN1* activity [34].

**Figure 8:**
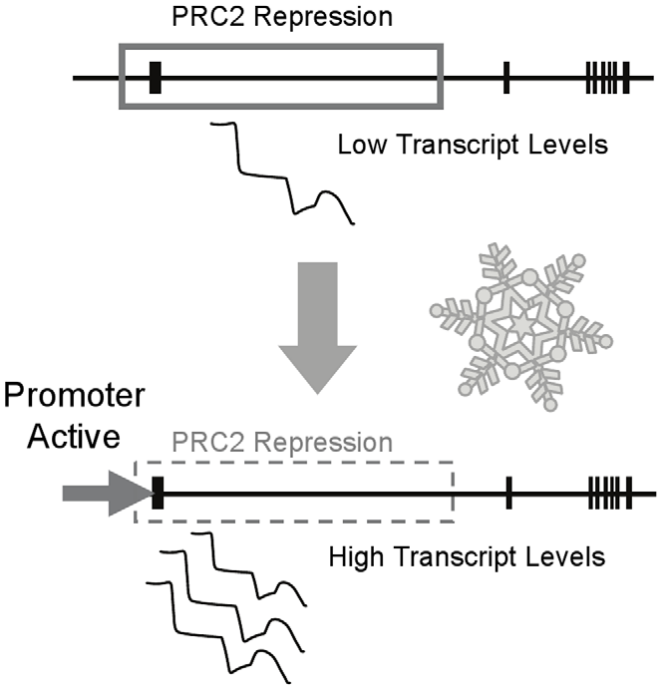
A model for transcriptional regulation of *VRN1*. Prior to winter, chromatin at the *VRN1* locus is maintained in an inactive state by histone modifications deposited by a plant Polycomb Repressor Complex 2 (PRC2), resulting in low transcript levels. When plants are exposed to prolonged cold (snowflake) the promoter of *VRN1* becomes more active, leading to increased transcription and higher steady state transcript levels. This triggers a change in the state of chromatin at the *VRN1* locus, with a shift towards an active state.

In addition to driving basal expression of a reporter gene in the shoot apex and developing leaves, a 2 kb segment from the promoter of *VRN1* was sufficient for induction of reporter gene expression by prolonged cold. Based on these observations, and those of previous studies [26]–[29], we suggest that the vernalization response of cereals depends largely upon two antagonistic regulatory mechanisms that control transcriptional activity of *VRN1*: activation of transcription by the promoter, which has a low basal activity but can be activated by cold, counteracted by constitutive repression through the first intron of the gene (Figure 8).

A key question is what mechanisms act at the promoter to mediate cold induction? The barley cultivar used in this study lacks *VRN2*, ruling out involvement of this gene. Similarly, the first intron is not present in the *P_VRN1_:GFP* reporter gene fusion, which is low-temperature responsive (Figure 3A), consistent with previous suggestions that the intron is not critical for cold induction [27], [31]. The *VRN1* promoter contains the putative VRN box, a region mutated in some active *VRN1* alleles of wheat [23]. An insertion in this promoter element (the *Vrn-A1a* allele described by Fu *et al.* [24]) did not disrupt low-temperature induction of *VRN1* (Figure 7), however, suggesting that while this region can influence basal expression of *VRN1* it is not critical for low-temperature induction. Presumably mutations in this region affect a transcriptional repression mechanism that acts separately to any mechanisms that mediate low-temperature induction.

The promoter of *VRN1* contains sequence motifs that might be targeted by low-temperature responsive transcription factors, including putative CBF and myc recognition sites (Figure 6). Binding of low-temperature responsive transcription factors, such as CBFs or the myc INDUCER OF CBF EXPRESSION1 (ICE1), to these putative binding sites might play a role in inducing expression of *VRN1* during vernalization. The promoter of the barley *VRN1* gene also contains binding sites for B-ZIP transcription factors that might mediate long-day activation of *VRN1* in vernalized plants or in some early flowering genotypes [36]. The potential role of these putative regulatory elements will be examined in future studies.

Another potential regulatory mechanism identified in the 5′ region of *VRN1* is a small upstream open reading frame (uORF). uORFs are widespread in mammalian genes and typically reduce protein expression from larger downstream open reading frames by 30–80% [37]. In humans, the presence of a uORFs in a gene can vary between individuals, causing variation in protein expression from downstream open reading frames and this is the basis for some diseases [37]. The uORF in the 5′ of the *VRN1* gene is conserved amongst *VRN1* orthologues in temperate cereals and related grasses (Figure 6B). The uORF was not required for the transcriptional response to prolonged cold, since it was not included in the low-temperature responsive *P_VRN1_:GFP* construct, but could potentially influence post-transcriptional regulation of the endogenous *VRN1* gene. In some early flowering accessions of the wild wheat *T. timopheevi* the uORF is deleted (Figure S3, [38]), although it is unclear whether this contributes to natural variation in flowering behaviour of this wild wheat.

In summary, we have shown that the promoter of *VRN1* is able to drive basal expression of the GFP reporter gene in the leaves and shoot apex of transgenic barley plants and is sufficient for induction of reporter gene expression by prolonged cold. Understanding the mechanisms that act at the promoter of *VRN1* to mediate the transcriptional response to prolonged cold of this central regulator of flowering can provide further insights into how vernalization induces spring flowering of economically important cereal crops.

## Materials and Methods

### Construction of *VRN1*-reporter gene fusion constructs

The *P_VRN1_:GFP* construct was generated by sub-cloning a 2 kb SpeI-NcoI *VRN1* promoter fragment from the Morex BAC clone 631P8 and ligating this to a *GFP*-nopaline synthase terminator cassette. The resulting promoter-reporter gene cassette was inserted into the pVEC8 T-DNA vector [39]. The *P_VRN1_EX1-2::GFP* construct was generated by first amplifying the promoter, first exon and a small segment of the first intron (5′-CACACCGCTCTACTAGTTC-3′ and 5′-GGCGCGCCCTGCAGGGAGCGCCAGCTCCGCC-3′) of *VRN1* from a winter barley (cv. Sonja). This was ligated to a second fragment from inside the first intron into the second exon (5′-GGCGCGCCCGATTATCAAAATGTACCA-3′ and 5′-CCCGGGTCACTTGAAACGAGAACC-3′), to generate a construct with 2 kb of promoter sequence, with the first and second (partial) exons separated by a 0.35 kb segment of the first intron. The resulting fragment was ligated to the GFP-nopaline synthase terminator, and inserted into pVEC8. The *VRN1::GFP* fusion was generated by fusing the 3′ UTR of VRN1, amplified from the Morex BAC 631P8 (5′-TTTCTAGACAGCCCATGTAAGCGTACTATTCAG-3′ and 5′-TTACTAGTAATGGCAGGTGTTCTGTTTGTTTATG-3′), to GFP. The GFP-UTR fragment was then ligated to a fragment amplified from the Morex BAC 631P8, from within the first intron to the last exon, minus the stop codon (5′-TTTCTAGACAGCCCATGTAAGCGTACTATTCAG-3′ and 5′-TTACTAGTAATGGCAGGTGTTCTGTTTGTTTATG-3′), with the open reading frames of *VRN1* and *GFP* in frame. Primers included compatible restriction sites to facilitate cloning. The resulting *VRN1-GFP-UTR* fragment was then ligated to the SpeI-NotI promoter fragment and the resulting reporter gene fusion was inserted into the pVEC8 T-DNA vector. The resulting *VRN1::GFP* fusion lacks most of the first intron (bp 2557 to 12214 relative to the Strider *VRN1* sequence) similar to the *HvVRN1-3* allele (bp 2968 to 11687).

### Barley Transformation

Barley transformation was performed by Agrobacterium transformation of excised embryos [40], [41], with cv. Golden Promise, which flowers without vernalization and is daylength insensitive (genotype *HvVRN1-1*, *ΔHvVRN2, ppd-H1*). Expression analysis was performed with plants from the T1 generation. Plants were screened for the presence of transgenes using primers that amplify the hygromycin selectable marker gene (5′-AAAAGCCTGAACTCACCGC-3′ and 5′-TCGTCCATCACAGTTTGCC-3′).

### Gene expression analysis

Seeds were germinated in soil and grown in darkness in foil covered pots at 20 degrees or 4 degrees. RNA was extracted from seedlings with Spectrum Plant Total RNA Kit (Sigma Aldrich, www.sigma-aldrich.com) following manufacturer's instructions. RNA was treated with 10 units of RQ1 RNase-free DNase (Promega, Madison, WI) for 15 min at 37°C. Total RNA (5 µg) was reverse-transcribed with Super Script III reverse transcriptase (Invitrogen, www.invitrogen.com), according to manufacturer instructions. qRT-PCR was performed in a Rotorgene Q real-time PCR cycler (Qiagen; www.qiagen.com) as described previously [20].

The following primer sets were used for quantitative RT-PCR: *HvVRN1*: 5′-GGAAACTGAAGGCGAAGGTTGA and 5′-TGGTTCTTCCTGGCTCTGATATGTT-3′, *P_VRN1_:GFP* 5′-TACAACTACAACAGCCACAAC-3′ and 5′-GCTTCTCGTTGGGGTCTTTG-3′, *P_VRN1_EX1-2::GFP* 5′-AGAACAAGATCAACCGCCA-3′ and 5′-ATCGCCCTCGCCCTCGCCGG-3′, *VRN1::GFP* 5′-CGCAGATACCAGCAATCACCCAG-3′ and 5′-ATCGCCCTCGCCCTCGCCGG-3′. For quantification of expression of the A genome copy of the *VRN1* gene from bread wheat the homeoallele specific primers 5′-CAGCCTGGTGTATGTTGCGGTTGC-3′ and 5′-ATTACTCGTACAGCCATCTCAGCC-3′ were used. The expression of each gene was normalized to *ACTIN* using the Rotorgene software package (Qiagen, www.qiagen.com), which takes amplification efficiency of each primer set into account for quantification calculations. *ACTIN* primers have been described previously [19]. All data presented are the average of mRNA levels from three biological repeats, unless stated otherwise, with error bars representing the standard error of the mean.

### Rapid Amplification of cDNA ends (RACE)

5′ RACE was carried out with the FirstChoice RLM-RACE kit from Ambion (Ambion; www.invitrogen.com/ambion) following manufacturer′s instructions with total RNA from Golden Promise seedlings grown under control conditions or Golden Promise seedlings grown to an identical stage of development at 4 degrees (28 days). Nested PCR was performed with the 5′RACE Outer Primer provided by the kit and the gene specific VRN1-R Outer primer (5′-GCATTCGTGCATAAGTTGGTTC-3′) and for the Inner 5′ RLM-RACE PCR we used the 5′RACE Inner Primer and the gene specific VRN1-R Inner primer (5′-TATTGTCTCAACCTTCGCCTTC-3′). PCR products were sequenced after ethanol precipitation.

### Confocal microscopy

Barley apices and leaf primordia were dissected under a binocular dissecting microscope and mounted in water. The plant material was then observed in Leica SP2 confocal laser scanning microscope (Leica Microsystems, Sydney, Australia) equipped with a standard Ar 488 laser excitation. Excitation was at 488 nm, and emission was collected between 500 and 550 nm. Chlorophyll autofluorescence was collected between 650 and 720 nm. Nuclear localisation was confirmed by co-localization with 4′,6-diamidino-2-phenylindole (DAPI) stain (Figure S4). Spikes were fixed in 4% paraformaldehyde in 50 mM phosphate buffer, pH 7.2, overnight, then rinsed in buffer and stained with 5 ug/ml DAPI for 30 sec. After mounting in fresh buffer, GFP was detected with 488 nm excitation, and emission from 505–550 nm collected. DAPI was then detected with 405 nm excitation, and emission from 415–485 nm collected using a Leica SP2 confocal microscope in sequential scanning mode to avoid bleedthrough of DAPI fluorescence into the GFP emission channel.

### Production of near-isogenic wheat lines

Wheats carrying different *VRN1* alleles were crossed to the Australian wheat cultivar Sunstate (*PPD-D1* insensitive allele, *VRN-A1* wildtype, *VRN1-B1* intron deletion and *VRN-D1* intron deletion) and then backcrossed a further 4 generations before homozygous plants were selected for contrasting *VRN1* genotypes. The donors of different *VRN1* alleles were: AUS7374 for Langdon *VRN1* A genome allele with an intron deletion, AUS 2380 “Extra Early Blackhull” for recessive B and D genome alleles or AUS1499 for the *VRN*-*A1a* promoter insertion allele.

## Supporting Information

Figure S1 **Low-temperature induction of the** ***VRN1::GFP*** **construct.**Transcript levels for the *VRN1::GFP* fusion in transgenic seedlings. Expression was assayed in control seedlings, germinated at normal glasshouse temperatures (20 degrees for 4 days), and compared to seedlings germinated and grown to an identical stage of development at low temperatures (4 degrees for 28 days). Expression levels were assayed in two independent transgenic lines. Error bars show standard error. * indicates P<0.05.
(TIF)

Figure S2 **Sequence motifs in the** ***VRN1*** **promoter.**The promoter sequence of the barley *VRN1* gene showing potential transcription factor recognition sites and other putative regulatory sequences. The transcriptional start site, identified by 5′RACE is also indicated (TSS); this did not vary with different temperature treatments.
(DOC)

Figure S3 **Deletion of the 5**′ **small open reading frame from the** ***VRN1*** **gene of a wild wheat.**Comparison of the A genome *VRN1* gene from hexaploid wheat (*VRN-A*1, Genbank AY747600.1) with the sequence of the *VRN1* gene from *Triticum timopheevi* (Genbank GQ451763). The VRN box is shaded grey. The putative CARG box is shaded black and the region that encodes the small upstream open reading frame is shown in bold text and boxed.
(DOC)

Figure S4 **Co-localisation of DAPI staining and VRN1::GFP within cells of the developing inflorescence.**Localization of DAPI staining compared to VRN1::GFP signal in the developing glume of a barley inflorescence. Double headed arrow shows DAPI and GFP signal in the same nucleus.
(TIF)
