# Supplementary figures and images for: The Promoter of the Cereal VERNALIZATION1 Gene Is Sufficient for Transcriptional Induction by Prolonged Cold
Source: PLoS One. 2011 Dec 29;6(12):e29456. doi: 10.1371/journal.pone.0029456 (PMC3248443; doi:10.1371/journal.pone.0029456)

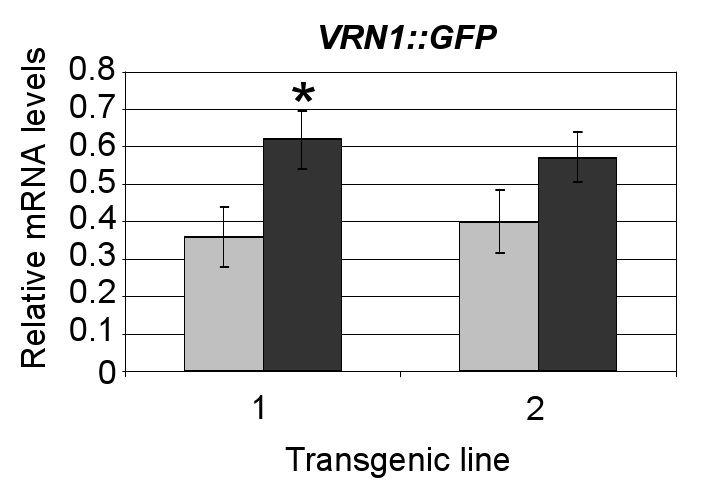

Supplement: Figure S1 — Low-temperature induction of the VRN1::GFP construct.Transcript levels for the VRN1::GFP fusion in transgenic seedlings. Expression was assayed in control seedlings, germinated at normal glasshouse temperatures (20 degrees for 4 days), and compared to seedlings germinated and grown to an identical stage of development at low temperatures (4 degrees for 28 days). Expression levels were assayed in two independent transgenic lines. Error bars show standard error. * indicates P<0.05. (TIF) [file pone.0029456.s001.tif]

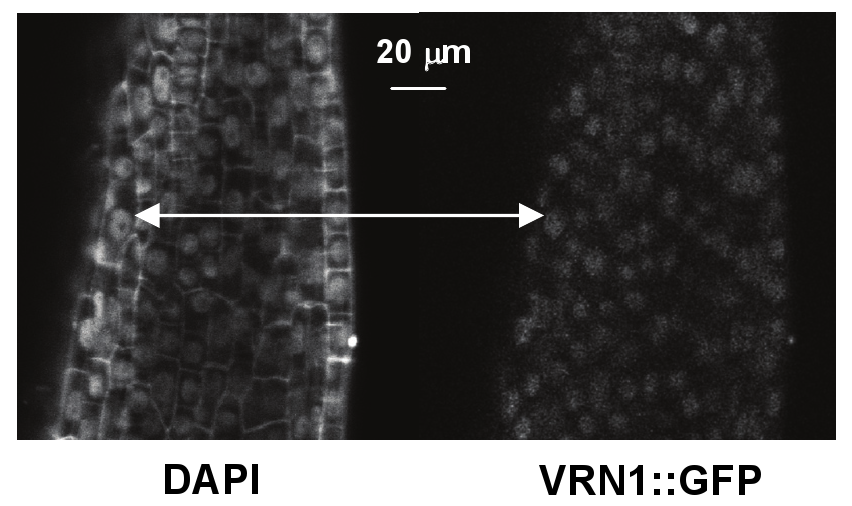

Supplement: Figure S4 — Co-localisation of DAPI staining and VRN1::GFP within cells of the developing inflorescence.Localization of DAPI staining compared to VRN1::GFP signal in the developing glume of a barley inflorescence. Double headed arrow shows DAPI and GFP signal in the same nucleus. (TIF) [file pone.0029456.s004.tif]
