# Supplementary material for: The Promoter of the Cereal VERNALIZATION1 Gene Is Sufficient for Transcriptional Induction by Prolonged Cold
Source: PLoS One. 2011 Dec 29;6(12):e29456. doi: 10.1371/journal.pone.0029456 (PMC3248443; doi:10.1371/journal.pone.0029456)
Supplement: Figure S2 — Sequence motifs in the VRN1 promoter.The promoter sequence of the barley VRN1 gene showing potential transcription factor recognition sites and other putative regulatory sequences. The transcriptional start site, identified by 5′RACE is also indicated (TSS); this did not vary with different temperature treatments. (DOC) [file pone.0029456.s002.doc]

-2100 -2090 -2080 -2070 -2060

SPE1.|....|....|....|....|....|....|....|....|....

**CTAGT**TCCCAATTTAAGAGTGGATTATTTGATCACGGGATCATCTATTCC

-2050 -2040 -2030 -2020 -2010

|....|....|....|....|....|....|....|....|....|....

CTATAAAACTTTGAGATGTTTTCTATGAAGTATGGTTTACACAGTATATC

-2000 -1990 -1980 -1970 -1960

|....|....|....|....|....|....|....|....|....|....

TGATCTAAATATTGGTTTTGCATTCTTGGAAACACACTGCAAAGTTTAGG

-1950 -1940 -1930 -1920 -1910

|....|....|....|....|....|....|....|....|....|....

GATGCTCATGTTTGTATCTTTTCAATCATAGAGATATACTCCCTCCGTCC

-1900 -1890 -1880 -1870 -1860

|....|....|....|....|....|....|....|....|....|....

GTCAAAAACTGTGCATCTAACAGTATATTTTTTTTCCATAAAGAGTGTAC

-1850 -1840 -1830 -1820 -1810

|....|....|....|....|....|....|....|....|....|....

ATCTACATTTTCTATGCGCTTAGCTTTTAATTTAACCGGTATTAAGTGAC

-1800 -1790 -1780 -1770 -1760

|....|....|....|....|....|....|....|....|....|....

TAATGTAGTAACTTGTGCTCTAGCTATTGGCTGCATGCCTAACCTTAATT

-1750 -1740 -1730 -1720 -1710

|....|....|....|....|....|....|....|....|....|....

ACTGCATGCAGCAACGTTCATTTAATCTTCTTTTCTCAATGGTTGTATGT

-1700 -1690 -1680 -1670 -1660

|....|....|....|....|....|....|....|....|....|....

ACACATAAGTCTGTTATTTTTGTACAATAATTATGTCATGGAGGTCTTTC

-1650 -1640 -1630 -1620 -1610

|....|....|....|....|....|....|....|....|....|....

TTGGTCTATGTGCAAATCTCTGTATGCACGGTTTTTGGCGGACGGAGGGA

-1600 -1590 -1580 -1570 -1560

|....|....|....|....|....|....|....|....|....|....

GTACACGACATATTCACAAGCAAAAAAGAAAAAGTTGGACATGGCATATT

-1550 -1540 -1530 -1520 -1510

|....|....|....|....|....|....|....|....|....|....

CGCAAAAAAAGAAATAAGAAAAGAGAAGAGATGTGCATGGCATGATTGTG

-1500 -1490 -1480 -1470 -1460

|....|....|....|....|....|....|....|....|....|....

AAACCTATCCTCATCGATCGTAAAAAGCGGTATTGGTATTTTTAGGGGAA

-1450 -1440 -1430 -1420 -1410

|....|....|....|....|....|....|....|....|....|....

TAAAGAGCGCTATTGGTGGTTGCTGGTTACATCGAATGGTTGCAAAGTGG

-1400 -1390 -1380 -1370 -1360

|....|....|....|....|....|....|....|....|....|....

TCATTTGACAAGCACGCATGTGGGCTTTATCTGTTTGTCATCATAAACGA

-1350 -1340 -1330 -1320 -1310

|....|....|....|....|....|....|....|....|....|....

TAAATGGGTCAAGATTCACAGATCAGAGAATACGTCGTCAAAGTCTCGAA

-1300 -1290 -1280 -1270 -1260

|....|....|....|....|....|....|....|....|....|....

CACTTCACTGCGTGGTCTACTGGGCCCAATAGCAGTTTTAGTGAACAAAC

-1250 -1240 -1230 -1220 -1210

|....|....|....|....|....|....|....|....|....|....

CATTTTAGTGCTGCAATAGTTTCAGCACGATCCTTTCCTTTGTACCGAGA

-1200 -1190 -1180 -1170 -1160

|....|....|....|....|....|....|....|....|....SspI.

AGCCGGTCACCGCAGATATATCACATGCACTTTCCGCATTTTTTT**AATAT**

-1150 -1140 -1130 -1120 -1110

|....|....|....|....|....|....|....|....|....|....

**T**ATATCACATGCACAGTTCTACAAGACATACACAGAGCGTTTATTTTTTT

-1100 -1090 -1080 -1070 -1060

|....|....|....|....|....|....|....|....|....|....

TGTGAGAAGGAAAACACATGAGCCGTTTCAACATCGCATGATTCGACGCT

-1050 -1040 -1030 -1020 -1010

|....|....|....|....|....|....|....|....|..BssHII.

GGGCAACAGTGTATTGATGGGTGGAGGACTGGTCGGCGCACAC**GCGCGC**A

-1000 -990 -980 -970 -960

|....|....|....|....|....|....|....|....|....|....

CAGTACCCCTACTCCGGCGGGAGTATCTTCCATTCATTCCAGAAATACGC

-950 -940 -930 -920 -910

|....|....|....|....|....|....|....|....|....|....

GGGTCGGCCAAAAGTAGAAAAATACACTGCGCCGACCCAACCCACACGCA

-900 -890 -880 -870 -860

|....|....|....|....|....|....|....|....|....|....

GCAACGGTTCGCGTCAAAAGTCCAGCTCGCGTCAATCATGCACGCACACG

-850 -840 -830 -820 -810

|....|....|...BsrBI.|....|....|....|....|....|....

GTAGACGCGCTGC**GAGCGG**AGGCGGAACCCATCCGTGTCTGCCCGCCCGC

-800 -790 -780 -770 -760

|....|....|....|....|....|....|....|....|....|....

CCCGCAGCCGCCCTCCCAAACGGGACAAGCCGGGGCGGCCCAAAACGAGC

-750 -740 -730 -720 -710

|....|....|....|....|....|....|....|....|....|....

AAGGAAAGCAGCCTCCTACTGTGGCAGCCCGCCCCCACGACCACCATCTC

-700 -690 -680 -670 -660

|....|....|....|....|....|....|....|....|....|....

GCCTTCCATTTCCCTGGACGGACCAGAGCCGTCCCGAGCCGCCCCTGACC

-650 -640 -630 -620 -610

|....|....|....|....|....|....|....|....|....|....

TAGCCACCCAGCATTTCCTGTTTCGTCCCGCGCCGCCGTGACGTGACCGA

-600 -590 -580 -570 -560

|....|....|....|....|....|....|....|....|....|....

GAAAAGCAAAAGAGGAAAAAGCGAAAATGCTAAAGGAAAAAACTCTGCTC

-550 -540 -530 -520 -510

|....|....|....|....|....|.AvrII...|....|....|....

TTTTATTCCTTCTATATCTACTCCAG**CCTAGG**GTACACACTATATATATA

-500 -490 -480 -470 -460

|....|....|....|....|....|....|....|....|....|....

TATATATATATATATATATATATATATATATATATATATATATATATATA

-450 -440 -430 -420 -410

|....|....|....|....|....|....|....|....|....|....

AAAGTAGAAAAAAGAAGAAGAAAATGTTGCTCTACTGCTCTATGGTGTGG

-400 -390 -380 -370 -360

|....|....|....|....|....|....|....|....|....|....

GTTTGTGGCGAGAAAAAATGATTTGGGGAAAGCAATATGGGGGAGATTCG

-350 -340 -330 -320 -310

|....|....|....|....|....|....|....|....|....|....

CGCGTACGATCGTCCGACACGTCGACACGGGGCGGGCCCGCGGTGGGGCA

-300 -290 -280 -270 -260

|....|.. PST1 .|....|....|....|....|....|....|....

TCGTGTGG**CTGCAG**GACCGCGGGGCCCCGCGGCGCGGGCCGGGCCAATGG

-250 -240 -230 -220 -210

|....|....|....|....|....|....|....|....|....|....

GTGCTCGACAGCGGACATGCCCCAGACCAGCCCGGTATTGCATACCGCGC

-200 -190 -180 -170 -160

|....|....|....|....|....|....|....|....|....|....

TCGGGGCCAGATCCCTTTAAAACCCCCTCCCGTCGCCCTGCCGGAACCCT

-150 -140 -130 -120 -110

|.+TSS....|....|....|....|....|....|....|....|....

CATTTGGCCATCCCCTCTCCCCTCCCACTTCACCCAACCACCTGACAG**CC**

-100 -90 -80 -70 -60

NCO1..|....|....|....|....|....|....|....|....|....

**ATGG**CTCCGCCACCTCGCCTCCGCCCGCGCCTCTCGGAGTAGCCGTCGCG

-50 -40 -30 -20 -10

|....|....|....|....|....|....|....|....|....|....

GTCGCTCGCTCGCTCGCTGCTGCCGGTGTTGGCCCGGTCCTCGAGCGGAG

0

|..

ATG

ICE1 CANNTG

BZIP ACGT

VRN BOX TTTAAAACCCCCTCCCG

ERE GCCGCC

Potential CBF sites

CRT/DRE CCGAC

CBF1* RCCGAC

Core Sequence GTCGAC

+TSS Transcriptional start site determined by 5’ RACE in control or prolonged cold treated seedlings.

*CBF1 sites based on Xue *Biochimica Biophysica Acta* 1577:63-72 (2002).
