# Supplementary material for: The Promoter of the Cereal VERNALIZATION1 Gene Is Sufficient for Transcriptional Induction by Prolonged Cold
Source: PLoS One. 2011 Dec 29;6(12):e29456. doi: 10.1371/journal.pone.0029456 (PMC3248443; doi:10.1371/journal.pone.0029456)
Supplement: Figure S3 — Deletion of the 5′ small open reading frame from the VRN1 gene of a wild wheat.Comparison of the A genome VRN1 gene from hexaploid wheat (VRN-A1, Genbank AY747600.1) with the sequence of the VRN1 gene from Triticum timopheevi (Genbank GQ451763). The VRN box is shaded grey. The putative CARG box is shaded black and the region that encodes the small upstream open reading frame is shown in bold text and boxed. (DOC) [file pone.0029456.s003.doc]

Figure S3

10 20 30 40 50 60

....|....|....|....|....|....|....|....|....|....|....|....|

VRN-A1 AAGGAAAAATTCTGCTCGTTTTTTTTCTCTGTGGTGTGTGTTTGTGGCGAGAGAAAATGA

GQ451763 AAGGAAAAATTCTGCTCGTTTTTTTGCTCTGTGGTGTGTGTTTGTGGCGAGAGAAAATGA

70 80 90 100 110 120

....|....|....|....|....|....|....|....|....|....|....|....|

VRN-A1 TTTGGGGAAAGCAAAATCCGGAGATTCGCACGTACGATCGTTCGACACGTCGACGCCCGG

GQ451763 TTTGGGGAAAGCAAAATCCGGAGATTCGCACGTACGATCGTTCGACACGTCGACGCCCGG

130 140 150 160 170 180

....|....|....|....|....|....|....|....|....|....|....|....|

VRN-A1 CGGGCCCGGGGTGGGGCATCGTGTGGCTGCAGGACCGCGGGGCCCCGCAAAGCGGGCCGG

GQ451763 CGGGCCCGGGGTGGGGCATCGTGTGGCTGCAGGACCGCGGGGCCCCGCAAAGCGGGCCGG

190 200 210 220 230 240

....|....|....|....|....|....|....|....|....|....|....|....|

VRN-A1 GCCAATGGGTGCTCGACAGCGGCTATGCTCCAGACCAGCCCGGTATTGCATACCGCGCTC

GQ451763 GCCAATGGGTGCTCGACAGCGGCTATGCTCCAGACCAGCCCGGTATTGCATACCGCGCTC

250 260 270 280 290 300

....|....|....|....|....|....|....|....|....|....|....|....|

VRN-A1 GGGGCCAGATCCCTTTAAAAACCCCTCCCCCCC---TGCCGGAATCCTCGTTTTGGCCTG

GQ451763 GGGGCCAGATCCCTTTAAAAACCCCTCCCCCCCCCCTGCCGGACCCCTCGTTTTGGCCTG

310 320 330 340 350 360

....|....|....|....|....|....|....|....|....|....|....|....|

VRN-A1 GCCATCCTCCCTCTCCTCCCCTCTCTTCCACCTCACGTCCTCACCCAACCACCTGATAGC

GQ451763 GCCATCCTCCCTCTCCTCCCCTCTCTTCCA--------CCTCACCCAACCACC-------

370 380 390 400 410 420

....|....|....|....|....|....|....|....|....|....|....|....|

VRN-A1 C**ATGGCTCCGCCGCCTCGCCTCCGCCTGCGCCAGTCGGAGTAG**CCGTCGCGGTCTGCCGG

GQ451763 -------------------------------------------CCGTCGCGGTCTGCCGG

430 440 450 460

....|....|....|....|....|....|....|....|....|...

VRN-A1 TGTTGGAGGGTAGGGGCGTAGGGTTGGCCCGGTTCTCGAGCGGAGATG

GQ451763 TGTTGGAGGGTAGGGGCGTAGGGTTGGCCCGGTTCTCGAGCGGAGATG
